# Transient Receptor Potential (TRP) Channels in the Pacific Oyster (*Crassostrea gigas*): Genome-Wide Identification and Expression Profiling after Heat Stress between *C. gigas* and *C. angulata*

**DOI:** 10.3390/ijms22063222

**Published:** 2021-03-22

**Authors:** Huiru Fu, Zexin Jiao, Yongjing Li, Jing Tian, Liting Ren, Fuqiang Zhang, Qi Li, Shikai Liu

**Affiliations:** 1 Key Laboratory of Mariculture, Ministry of Education, College of Fisheries, Qingdao 266003, China; fuhuiru@stu.ouc.edu.cn (H.F.); zexinjiao@foxmail.com (Z.J.); liyongjing@stu.ouc.edu.cn (Y.L.); tj2357@stu.ouc.edu.cn (J.T.); renliting@stu.ouc.edu.cn (L.R.); zhangfuqiang@stu.ouc.edu.cn (F.Z.); qili66@ouc.edu.cn (Q.L.); 2 Laboratory for Marine Fisheries Science and Food Production Processes, Qingdao National Laboratory for Marine Science and Technology, Qingdao 266237, China

**Keywords:** *Crassostrea gigas*, TRPs, phylogeny, gene expression, thermal stress

## Abstract

Transmembrane proteins are involved in an array of stress responses, particularly in thermo-sensation and thermo-regulation. In this study, we performed a genome-wide identification and characterization of the Transient Receptor Potential (TRP) genes in the Pacific oyster (*Crassostrea gigas*) and investigated their expression profiles after heat stress to identify critical TRPs potentially associated with thermal regulation. A total of 66 TRP genes were identified in the *C. gigas*, which showed significant gene expansion and tandem duplication. Meta-analysis of the available RNA-Seq data generated from samples after acute heat stress revealed a set of heat-inducible TRPs. Further examination of their expression profiles under chronic heat stress, and comparison between *C. gigas* and *C. angulata*, two oyster species with different tolerance levels to heat stress, led to the identification of TRPC3.6, TRPC3.7, and TRPV4.7 as important TRPs involved in thermal regulation in oysters. This work provided valuable information for future studies on the molecular mechanism of TRP mediated thermal tolerance, and identification of diagnostic biomarker for thermal stress in the oysters.

## 1. Introduction

Global climate change has driven environmental changes dramatically, including global temperature rise, shrinking ice sheets, and ocean warming (https://climate.nasa.gov, accessed on 20 January 2021). Global ocean warming is a critical indicator of the climate system. Surface temperature of the ocean has hit record high in 2020 and a continued increasing has been predicted [1]. Ocean temperature is a prominent external factor affecting biochemical and physiological performance of organisms, especially the aquatic ectotherms [2]. Mollusks are ecologically important components of aquatic ecosystems [3]. Of which, oysters are a major group of marine mollusks and have well adapted to estuarine and intertidal environment [4]. However, the oysters have been encountering mass mortality during summer in recent years. Elevated water temperature has been considered to be one of the major causes for mass summer mortality in oyster aquaculture world-wide [5,6,7].

It is essential to unravel molecular mechanism for oysters to survive in elevated temperature toward studying response and adaptation to global warming. The alterations of gene expression under heat stress have been extensively studied in oysters, and several important genes potentially involved in stress tolerance have been identified, with much focus on heat-shock proteins [8,9,10,11]. However, can oysters sense temperature and how to sense and respond to temperature change according to the environmental and physiological status in order to survive still remains largely unknown.

Transient receptor potential (TRP) channels are cation-permeable six transmembrane ion channels that can be activated by specific stressors and are involved in stress-sensation and stress-regulation [12]. Numerous TRP genes have been identified in diverse organisms since the first TRP was reported in Drosophila [13]. The TRP superfamily is broadly divided into seven subfamilies, including TRPC (Canonical), TRPV (Vanilloid), TRPM (Melastatin), TRPA (Ankyrin), TRPN (No mechano-potential), TRPP (Polycystin) and TRPML (Mucolipin). TRPs are involved in various processes of sensory reception, including thermoreception, chemoreception, mechanoreception, and photoreception [14,15,16].

Some TRPs are capable of detecting and responding to hot and cold. For instance, TRPV1-4, TRPA1, and TRPM8 are expressed in sensory neurons and can be gated by changes in temperature, which are considered as thermosensors [17]. Some TRP channels also act as polymodal sensors that are activated by other physical (e.g., osmotic pressure, mechanical, voltage) and chemical stimuli. For example, TRPM2 is one of the oxidative-stress-dependent cation channels, which is activated by reactive oxygen species (ROS) or hydrogen peroxide (H_2_O_2_) [18,19,20]. Previous studies also revealed the involvement of TRPs in other biological processes. TRPM2 is critically involved in diverse physiological processes not only temperature sensing but also apoptosis and immune response [21,22]. TRPCs are involved in control of energy homeostasis in which activation of TRPC channel is connected to PI3K-AKT signaling pathways [23]. TRPC3 channels are required in central glucose sensing and regulation of energy balance by mediating the effect of increased glucose on glucose-excited neurons through a mechanism that depends on ROS production [24].

Systematic identification of known thermo-sensory TRPs and profiling their expression in response to heat stress in oysters would provide valuable information to the research community. Essential genomic resources are publicly available, including whole genome sequences [11] and transcriptome datasets [9,25,26,27], toward systematic identification and annotation of TRPs in *C. gigas*. In the present study, we performed an extensive data mining of the available genome and transcriptome datasets and identified a complete set of 66 TRP genes in *C. gigas*. Expression profiles of these TRPs as determined from the available RNA-seq datasets allowed identification of several heat inducible TRPs in the *C. gigas*. Furthermore, we compared their expression profiles upon chronic heat stress between *C. gigas* and *C. angulata*, two oyster species with contrasted thermal tolerance, which naturally inhabit the northern and southern intertidal areas across the China coastline [28,29]. We reasoned that comparison of expression profiles of the heat inducible TRP genes between the two oyster species with different thermal tolerance would allow identification of critical TRPs involved in thermal tolerance. Our work provides valuable information for future studies on the molecular mechanism of TRP mediated thermal tolerance, and offers new insights into global warming adaptation in marine mollusks.

## 2. Results

### 2.1. Genome-Wide Identification of TRP Genes in C. gigas

**Figure 1:**
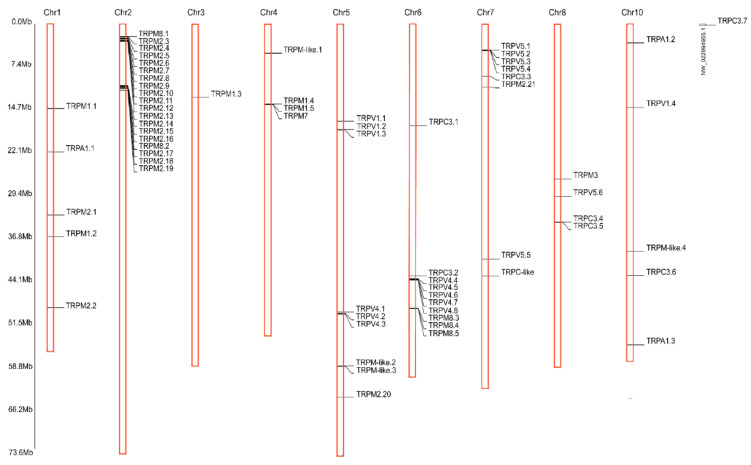
Identification of TRP genes and their distribution across chromosomes of *C. gigas*. The scale on the right is in million bases (Mb). Chromosome numbers are shown at the top of each vertical bar. Genomic locations of TRP genes are marked with the black lines.

A total of 66 TRP genes were identified in the *C. gigas* genome. Phylogenetic and syntenic analysis allowed correct annotation of these genes. Gene symbols used in this study followed the numerical designation, according to their locations on the chromosome. Notably, significant gene expansions were observed in several TRP subfamilies. For the tandem duplicates of a TRP gene, we assigned unique names according to their genomic location on the chromosomes. The detailed information of these 66 TRP genes, including unique gene names, sequence characteristics, chromosome location, and accession numbers were provided in Supplementary Table S1. Lengths of proteins encoded by these 66 TRP genes ranged from 345 to 1782 amino acids, with the transcript length ranging from 913 to 7990 nucleotides with or without 5′- and 3′- untranslated regions (UTRs). These TRP genes were located on nine chromosomes except for chromosome 9. Only one TRP (TRPC3.7) was identified on a scaffold sequence (NW_022994955.1). Notably, 19 TRP genes were clustered on the chromosome 2, while only one TRP gene was located on chromosome 3 (Figure 1).

### 2.2. Phylogenetic Analysis of C. gigas TRP Genes

**Figure 2:**
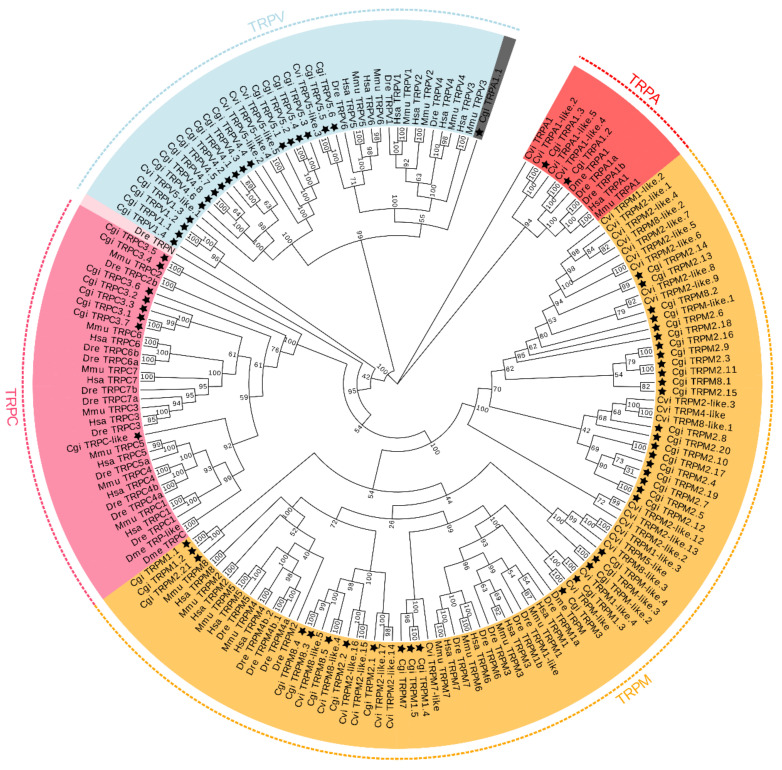
Phylogenetic analysis of TRP genes in *C. gigas*. The maximum-likelihood tree was inferred using the edge-linked partition model in IQ-TREE program. Numbers around the nodes correspond to bootstrap support values in percentages. Accession numbers for all sequences are provided in Table S1. Abbreviations: Hsa, *Homo sapiens*; Mmu, *Mus musculus*; Dre, *Danio rerio*; Dme, *Drosophila melanogaster*, Cvi, *Crassostrea virginica* and Cgi, *Crassostrea gigas*. The *C. gigas* TRPs are indicated by stars.

Phylogenetic analysis was conducted with TRPs identified from *C. gigas* and several other representative species. As shown in Figure 2, the 66 TRPs were divided into four subfamilies, including TRPA, TRPM, TRPC, and TRPV. Overall, most TRPs were clustered into their corresponding subfamilies containing counterparts from other species with strong bootstrap supports, except that TRPA1.1. TRPM2/8 and TRPM1/3/7 were separately clustered. TRPC1, TRPC2, TRPC4/5, and TRPC6/3/7 were grouped together to form separate clades, respectively. Notably, most of the *C. gigas* TRPVs were not clustered with those of vertebrates.

### 2.3. Syntenic Analysis of C. gigas TRP Genes

**Figure 3:**
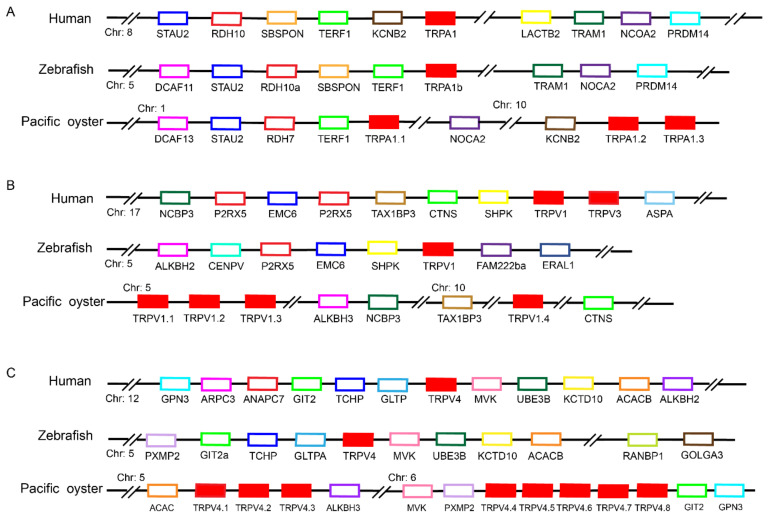
Syntenic analyses of (**A**) TRPA1, (**B**) TRPV1, and (**C**) TRPV4 in *C. gigas*. The TRP genes were highlighted in solid red box, and their neighboring genes were indicated by different color boxes with gene symbols. The syntenic information of TRPs for other species was from Genomicus (v96.01).

Syntenic analysis was conducted for several TRPs to provide additional evidence of orthology, confirming the results from the phylogenetic tree (Figure 3). For TRPA, especially TRPA1.1, the syntenic regions containing the TRPA gene were well conserved between the vertebrates and oysters. In addition, conserved syntenic blocks were identified for TRPV1 and TRPV4, which were not clearly described in NCBI (Supplementary Table S1). The TRPV1, ALKBH3 and NCBP3 were located on the same chromosome in *C. gigas*, although spanned long genomic distances. NCBP3 and ALKBH3 were co-located with TRPV1 on the same chromosome in human and zebrafish, respectively. Similarly, the oyster TRPV4 shared the same neighboring genes with human and zebrafish, including ACAC, ALKBH3, MVK, PXMP2, GIT2 and CPN3. The tandem duplicates of TRPV4 were clearly observed. According to the syntenic information, both *C. gigas* TRPV1 and TRPV4 were renamed after the nomenclature of vertebrate orthologues.

### 2.4. Copy Numbers of TRP Genes from Representative Vertebrates and Invertebrates

In order to gain insights into evolution of TRP genes, the copy numbers of TRPs in *C. gigas* were compared with those of other species, including *Homo sapiens*, *Mus musculus*, *Gallus gallus*, *Xenopus tropicalis*, *Danio rerio*, *Ciona intestinalis*, *Mizuhopecten yessoensis*, and *Crassostrea virginica* (Table 1). In general, only single copy of each TRP subfamily member exists in vertebrates such as *Homo sapiens*, *Mus musculus* and *Gallus gallus*. While the copy numbers of TRP in *C. gigas* are much larger than those of higher vertebrates. Among these TRP genes found in *C. gigas*, almost all TRPs were duplicated except for TRPM3 and TRPM7. Specially, TRPA1 had three copies, TRPC3 had seven copies, TRPM1 had five copies, TRPM2 had 21 copies, TRPM8 had five copies, TRPV1 had four copies, TRPV4 had eight copies, and TRPV5 had six copies. Overall, significant gene expansion occurred in oysters.

### 2.5. Expression Profiling of TRP Genes in C. gigas after Acute Heat Stress

The expression profiling of all the 66 *C. gigas* TRP genes in response to acute heat stress revealed that three TRP genes were both up-regulated and 20 were both down-regulated at 12 h post-stress at 30 and 35 °C (Supplementary Table S2). Of which, three TRP genes, TRPC3.6, TRPC3.7, and TRPV4.7, were significantly up-regulated after 12 h at 30 and 35 °C. In contrast, seven of the TRP genes were significantly down-regulated at both temperature stress, all of which belong to TRPM subfamily. Larger numbers of differentially expressed TRP genes were identified at 35 °C than that of 30 °C.

### 2.6. Mortality of the Two Oyster Species under Chronic Heat Stress

**Figure 4:**
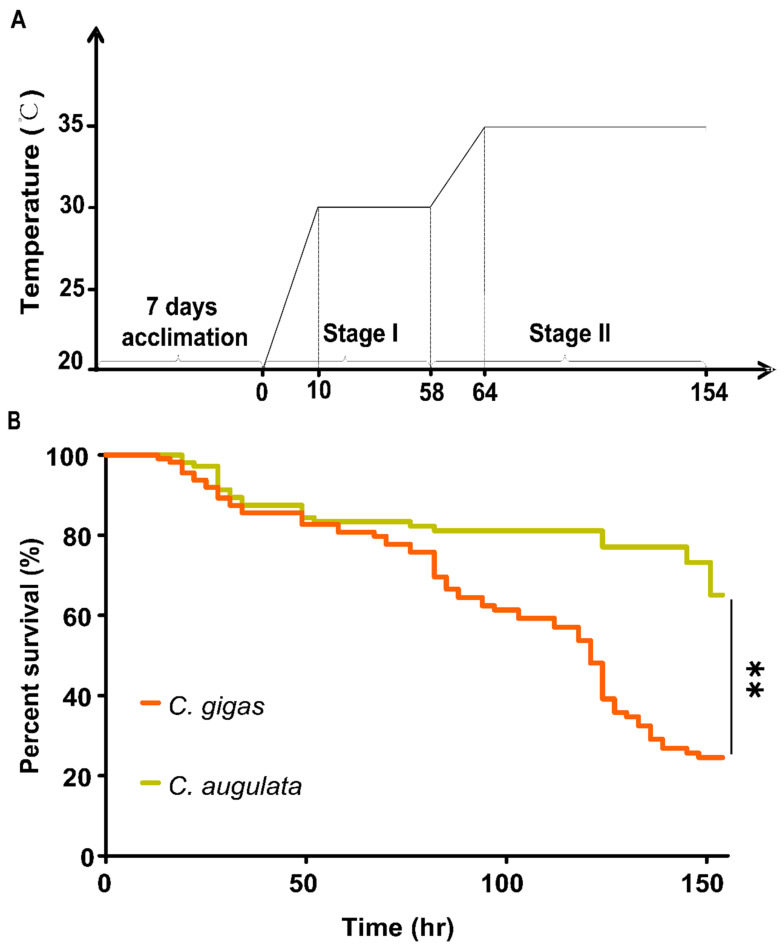
Chronic heat stress experiment and survival analysis. (**A**) Schematic presentation of the chronic heat stress experiment; (**B**) Mortality comparison between two oyster species. A number of 200 juvenile oysters per group were used for the experiment. ** indicates statistical significance (*p* < 0.01) (ANOVA).

In order to mimic the rising temperature leading to thermal stress in natural environments, we carried out a chronic increase in temperature in two stages. Water temperature was firstly elevated at a rate of 1 °C/h to 30 °C, which was held for two days, as stress stage I. Then, it was increased at a rate of 0.8 °C/h to 35 °C, which was held until the end of experiment, as stress stage II (Figure 4A). The oyster mortality was closely monitored during chronic heat stress experiment (Figure 4B). It is clear that *C. angulata* showed a significantly higher survival rate than *C. gigas* (*p* < 0.01) during the experiment, which was consistent with previous observations on their different tolerance to high temperature stress.

### 2.7. Relative Expression of TRPs in Response to Chronic Heat Stress

**Figure 5:**
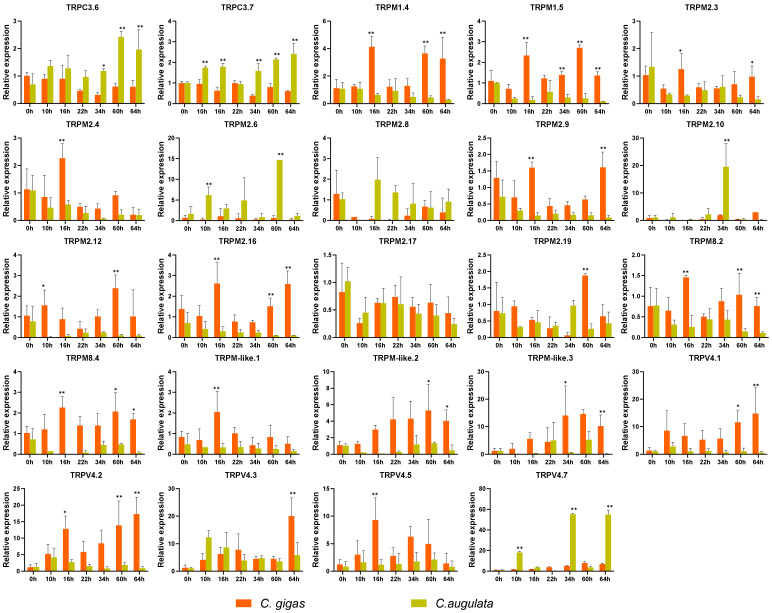
Relative expression of TRP genes between *C. gigas* and *C. angulata* after chronic heat stress. The Y-axis represents relative expression as normalized expression levels of the elongation factor 1 alpha (EF1α) gene. Each value of relative expression was shown as mean ± S.E. (*n* = 3). Asterisks indicate statistical significance (* indicates *p* < 0.05 and ** indicates *p* < 0.01) (ANOVA).

**Figure 6:**
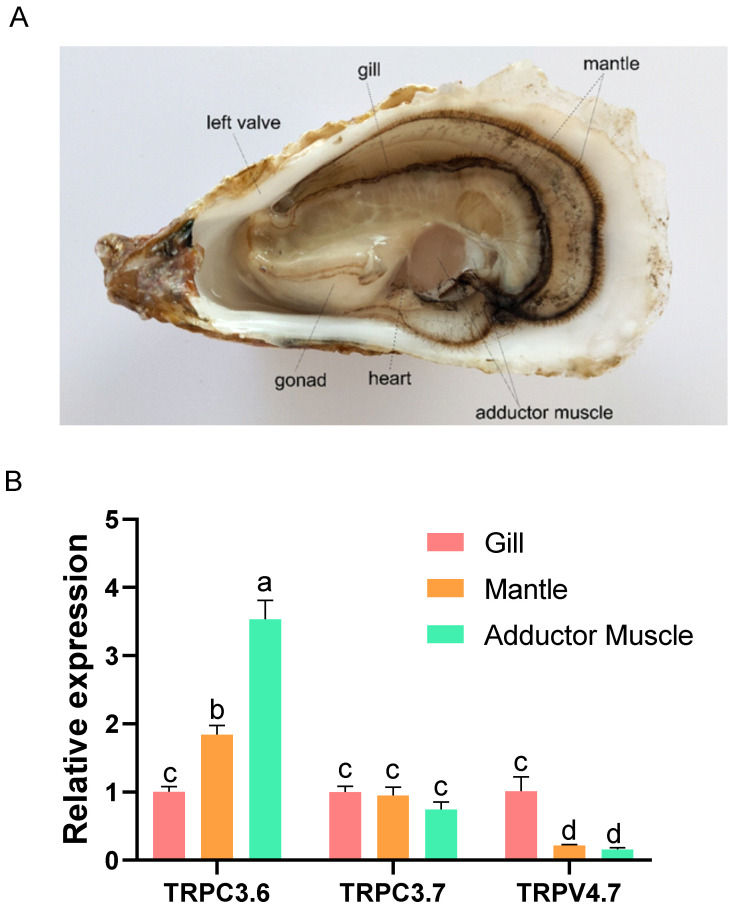
(**A**) The anatomy of the oyster; (**B**) Tissue distribution of selected TRP genes in oysters. The Y-axis represents relative expression as normalized expression levels of the elongation factor 1 alpha (EF1α) gene. Each value of relative expression was shown as mean ± S.E. (*n* = 3). Values in bars that share the same superscript letter are not significantly different (*p* > 0.05, Tukey’s test).

For the inducible TRP genes in response to acute heat stress, we further determined their expressions between *C. gigas* and *C. angulata* in response to chronic heat stress (Figure 5). Apparently, the relative expression of highly inducible genes (TRPC3.6, TRPC3.7, and TRPV4.7) was higher in *C. angulata* than in *C. gigas* during the whole heat stress experiment. In contrast, most of the TRPM genes were expressed higher in *C. gigas* that that in *C. angulata*, except for TRM2.6 and TRPM2.8. Expressions of highly inducible genes were then observed in all tested tissues (Figure 6A,B). Large differences were observed in the expression profiles of TRPC3.6 and TRPV4.7 among the tested tissues. TRPC3.6 was highly expressed in adductor muscle, while TRPV4.7 was highly expressed in gill. TRPC3.7 was expressed with no difference in all tested tissues.

### 2.8. Effect of Chronic Heat Stress on the Physiological Response of the Two Oysters

**Figure 7:**
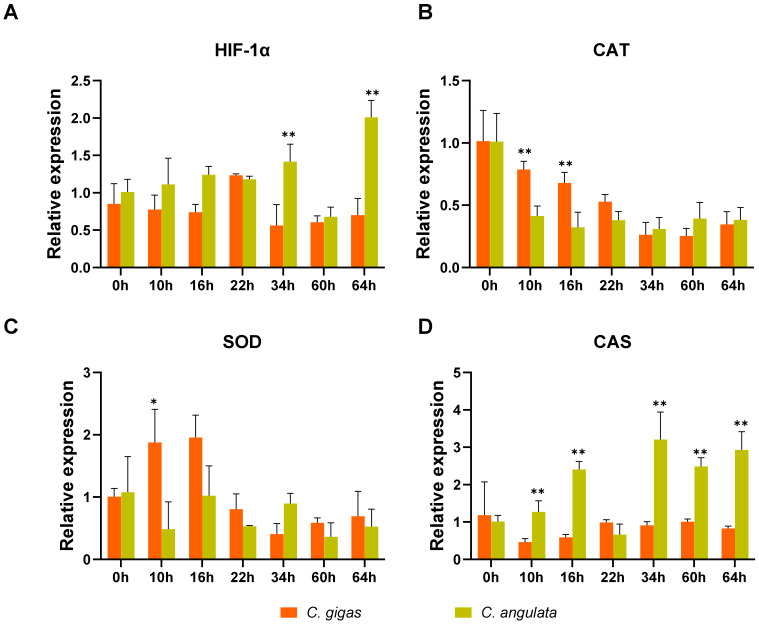
Effect of chronic heat stress on expression of (**A**) HIF-1α, (**B**) CAT, (**C**) SOD, (**D**) CAS in gill of two oyster species. Values were expressed as mean ± S.E (*n* = 3). Asterisks indicate statistical significance (* indicates *p* < 0.05 and ** indicates *p* < 0.01) (ANOVA). HIF-1α, hypoxia inducible factor-1α; CAT, catalase; SOD, superoxide dismutase; CAS, caspase.

In order to evaluate the physiological response to chronic heat stress of the oyster, we determined relative expression of four stress-related genes, including hypoxia inducible factor 1 α (HIF-1α), catalase (CAT), superoxide dismutase (SOD), and caspase (CAS) (Figure 7). HIF-1α and CAS were mainly induced in the latter stage, and relative expression of these two genes were higher in *C. angulata* than in *C. gigas*. In contrast, the expressions of CAT and SOD were being decreased with stress, and were higher in *C. gigas* than in *C. angulata*.

## 3. Discussion

Global warming has caused ocean warming and changes in the diversity of marine species, thus weaken the ability of the ocean and coast to provide critical ecosystem service (https://oceanfdn.org, accessed on 3 March 2021). Oysters are a group of marine mollusks which represent an essential component in coastal and marine ecosystems (http://www.fao.org, accessed on 3 March 2021). *C. gigas* and *C. angulata* naturally inhabit the northern and southern intertidal areas along China coastlines, showing a drastic difference in cope with temperature rising [28,29]. Their ability to survive in frequently fluctuated temperature makes them an ideal model for studying response and adaptation to ocean warming.

TRPs are important ion channels which can be gated by temperature, and play critical roles in thermo-sensation and thermo-regulation [12,16,30]. In this study, we performed exhaustive search in the *C. gigas* genome, conducted phylogenetic analysis, and determined the expression profiles of these genes by meta-analysis of RNA-seq datasets and qRT-PCR in oysters under both acute and chronic heat stress. These information should be useful for functional analysis and comparative genome analysis as well as for evolutionary studies in oyster species [31].

We identified a total of 66 TRP protein-coding genes (Supplementary Table S1). Most of the *C. giga* TRPs were clustered with their orthologs and formed their own clades with adjacent counterparts (Figure 2), suggesting that these genes were derived from lineage-specific events. Surprisingly, we did not observe the predicted classification of TRPA1.1 from the phylogenetic tree. Partial sequences used for alignment and phylogenetic analysis may account for this observation. Similar neighboring genes were found among Pacific oyster, human, and zebrafish, suggesting a conserved syntenic relationship for those ambiguously described genes in NCBI (Figure 3), which provided additional evidence for correct annotation of the TRP genes in *C. gigas*. The numbers of TRPs are highly variable among different vertebrates and invertebrates. Interestingly, the oysters possessed a significantly greater number of TRP genes compared with other organisms (Table 1). The mammalian TRP subfamily consists of more members, and each member contained only a single copy [12,32,33]. On the contrary, the oysters contained fewer family members, but almost all of the members are multiple copies, likely as a consequence of duplication events during evolution [11]. Remarkably, TRPM2 had 21 copies in *C. gigas*, followed by eight TRPV4 and seven TRPC3, which are much more than those in vertebrates. Genomic expansion was also observed in other stress responsible genes. The variation in gene copy numbers among species can be a reflection of regulatory variation affecting physiological difference in response to environmental fluctuations, especially temperature [11]. With oysters being the representative mollusks, these genes could be shared by other molluscan species (Table 1). Similarly, we speculated that genes absent in bivalves might undergo species-specific loss instead of the loss in all mollusks.

The involvement of TRPs in thermoreception has been revealed in some model organisms. To provide insight into functions of TRP genes in oyster, for the first time, we determined expression profiles of TRP genes by meta-analysis of available RNA-Seq data from acute heat stress [11]. We found 25 TRP genes were significantly regulated at 12 h after heat stress in *C. gigas* (Supplementary Table S2), suggesting their potential involvement in response to heat stress. For those heat inducible TRPs, we further determined their relative expression patterns under chronic heat stress. Drastically different response to high temperature was observed between the southern *C. angulata* and northern *C. gigas* when exposed to identical chronic high temperature stress, with a higher mortality in *C. gigas* than *C. angulata* (Figure 4A,B) which was consistent with the results from a previous study [34]. Consistently, the TRP genes were expressed differently in response to chronic heat stress between two oyster species (Figure 5). After exposure to chronic heat stress, highly inducible TRP genes were induced to much higher levels in *C. angulata* than in *C. gigas*. For the down-regulated TRPs, most were expressed lower in *C. angulata* than in *C. gigas*. Relative expression patterns of TRP genes in the *C. gigas* and *C. angulata* under same two-stage heat stress experiment conditions demonstrated that the regulation of TRP genes expression was species-specific and time dependent. A striking observation was that TRPC and TRPV4.7 were extremely up-regulated in *C. angulata* than in *C. gigas* suggesting its involvement in conferring heat tolerance in the oyster. The two up-regulated TRPs showed tissue-specific expression patterns, with TRPC3.6 being highly expressed in adductor muscle, while TRPV4.7 being highly expressed in gill (Figure 6B). TRPC3 channels are novel players in central glucose sensing and regulation of energy balance, mediating the effect of increased glucose on neurons through a mechanism that depends on ROS production [24]. Previous studies have also related TRPCs to PI3K-AKT signaling pathways [35]. AKT was reported increased expression under hypoxia conditions in the smooth muscle of *C. gigas*, involving in metabolism of muscle protein [36,37]. In terms of that, the inducible expression of TRPC3.6 in adductor muscle as we observed in this work may indicate its functional role in energy regulation in response to heat stress and oxidative damage. The responses of TRPV4 to heat stress show desensitization on repeated heat treatment [38,39]. In this work, TRPV4.7 was found to be very active under heat stress. In *C. gigas*, TRPV4.7 was dramatically up-regulated at early stage of heat stress, then returned to normal level quickly. The investigation of TRPV4 expression and its potential role in oxidative stress-induced cell damage shows that distinct differences in vulnerability to different forms of oxidative stress, suggesting the specific involvement of this channel in oxidative stress-induced cell damage [40]. Acute and chronic heat stress causing oxidative damage have also been reported in *C. gigas* [41]. The altered expression pattern of this gene may indicate its critical role in response to heat and heat-related stresses. Additionally, the induced expression of TRPV4.7 in the gill of *C. gigas* also suggested its important role in response to heat stress as oyster gill is the first line of defense against environmental stressors, which has been reported to be perturbed by thermal stress [42].

The protection against apoptosis and mitochondrial ROS through inhibition of TRPM2 in several types of neurons was recently reported [43]. In this study, most TRPM subfamily genes were significantly down-regulated after acute heat stress (Supplementary Table S2). The relative expression levels of these TRPM genes, especially TRPM2, were higher in northern *C. gigas* than those in southern *C. angulata* (Figure 5). This may imply a critical role of TRPM2 in maintaining dynamic equilibrium of ROS response and apoptosis in *C. gigas*. Since temperature increasing tends to aggravate hypoxia and its consequences, including generation of ROS [44,45,46,47], we measured the relative expression of several stress-related genes under chronic heat stress. The expression profiles of HIF-1α and antioxidant enzyme (CAT and SOD) indicated hypoxia and oxidative stress may have occurred. In addition, the caspase is highly induced in *C. angulata*, which may be related to alteration of TRPM2.

This is the first report on investigation of gene families involved in thermosensation in marine mollusks. The relationship between TRP genes expression and the survival of oysters under heat stress implying a potential role of these genes in thermal response in oysters, although we did not demonstrate the activation of these channels by detect electric current directly [38,39]. We hypothesized that expressions of TRP genes were the physiological basis of oysters in response to temperature change. Although further studies are warranted to explore the mechanisms of the TRP genes in heat tolerance, the altered expression profiles of TRPs during the acute and chronic heat stress, and drastic difference in expression levels between the two oyster species with contrasted heat tolerance capability suggested important roles of these TRPs in oysters for their adaptation to environments with highly frequent heat stress.

## 4. Materials and Methods

### 4.1. Identification of TRP Genes in C. gigas

To identify the full set of TRP genes in *C. gigas*, we downloaded the TRP protein sequences from several representative invertebrates, including *Branchiostoma belcheri*, *Ciona intestinalis*, *Acanthaster planci*, *Strongylocentrotus purpuratus*, *Drosophila melanogaster*, *Bombyx mori*, *Crassostrea virginica*, *Mizuhopecten yessoensis*, *Caenorhabditis elegans*, and *Exaiptasia pallida*, and several representative vertebrates, including *Homo sapiens*, *Mus musculus*, *Bos taurus*, *Meleagris gallopavo*, *Gallus gallus*, *Chrysemys picta bellii*, *Anolis carolinensis*, *Xenopus laevis*, *Xenopus tropicalis*, *Pangasianodon hypophthalmus*, and *Danio rerio*. The detailed information of these TRPs were provided in Supplementary Table S3. These sequences were downloaded from NCBI (https://www.ncbi.nlm.nih.gov, accessed on 3 March 2021) and Ensembl (http://grch37.ensembl.org/index.html, accessed on 3 March 2021) databases and used as queries to search against the reference genome *C. gigas* in the NCBI (Assembly: cgigas_uk_roslin_v1, with accession of GCA_902806645.1) using BLAST program with the E-value of 1E-10. The identified TRP-related sequences were further manually curated, followed by functional domain analysis using Conserved domain Search program (https://www.ncbi.nlm.nih.gov/Structure/cdd/wrpsb.cgi, accessed on 3 March 2021). The identifies of predicted TRP proteins from *C. gigas* were further confirmed by protein BLAST (BLASTP) [48] against NCBI non-redundant (Nr) protein sequence database. The distribution of the identified TRP genes on the chromosomes were drawn using TBtools [49].

### 4.2. Phylogenetic and Syntenic Analysis

Phylogenetic analysis was conducted with all the protein sequences of TRP genes identified from *C. gigas* and those from several other representative species. Multiple sequence alignments were performed with MAFFT [50] and the maximum likelihood phylogenetic tree was constructed using IQ-TREE [51,52] with bootstrap test of 1000 replicates. Dendrograms were created and colored using EvolView (https://evolgenius.info//evolview-v2/#login, accessed on 3 March 2021). For several TRPs with ambiguous identifies based on phylogenetic analysis, further syntenic analysis was conducted to provide additional evidence of orthology for the correct annotation. The neighboring genes of relevant TRP genes in human and zebrafish were determined based on the Genomicus database v96.01 [53]. For the *C. gigas*, neighboring genes of relevant TRPs were identified based on the latest genome annotation and BLAST search.

### 4.3. Expression Profiles of TRP Genes in C. gigas after Acute Heat Stress

Meta-analysis of RNA-seq datasets from public database was conducted to determine the expression profiles of TRP genes after acute heat stress. The Illumina-based RNA-Seq reads were obtained from a previously published study [11]. A total of three groups of oysters were exposed to 20, 30, and 35 °C, respectively. The gill tissues were collected at 12 h for RNA sequencing. The RNA-seq datasets have been deposit in NCBI sequence read archive (SRA) with the accessions of SRR334265 (20 °C group), SRR334267 (30 °C group), and SRR334268 (35 °C group). The 20 °C group was used as control to identify genes that were differentially expressed in response to acute heat stress. The RNA-Seq reads were mapped to the latest genome assembly (Assembly: cgigas_uk_roslin_v1, with accession of GCA_902806645.1) followed by differential expression analysis using HiSAT2-StingTie-Bowgown pipeline [54]. Mapping parameters were set as at least 95% of the reads in perfect alignment and maximum of two mismatches being allowed. The number of mapped reads for each transcript was counted and normalized to calculate Fragments Per Kilobase of transcript per Million mapped reads (FPKM). The proportions-based Kal’s test was performed to identify the differentially expressed genes comparing each time point sample with control sample. Transcripts with absolute fold change values of greater than two, total reads number more than five, and statistical significance level *p*-value < 0.05 after multiple testing correction, were identified as differentially expressed genes.

### 4.4. Chronic Heat Stress of C. gigas and C. angulata

The chronic heat stress experiment was performed using 200 juvenile *C. gigas* (6-month old) collected from a commercial farm in Weihai (Shandong, China) and 200 similarly sized wild *C. angulata* collected from Ningbo (Zhejiang, China) in 2018. Both groups of oysters were acclimated at temperature of 20 ± 1 °C for one week before experiment. Oysters were fed with Chlorella powder ad libitum twice a day during acclimation. Oysters were transferred to tanks which were set up with a constant flow system with fresh water of 20 ± 1 °C for experiment. Water temperature in the treatment tank was elevated at a rate of 1 °C/h to 30 °C, which was held for two days as stress stage I, and then increased at a rate of 0.8 °C/h to 35 °C, which was held until the end of experiment as stress stage II. Throughout the course of the experiment, oyster mortality was closely monitored and dead oysters were removed timely. Samples were collected at seven time points (0, 10, 16, 22, 34, 60, and 67 h) during experiment. At each time point, gill tissue from six oysters were dissected and pooled into three replicates (two oysters/replicate), flash-frozen in liquid nitrogen and stored at −80 °C until RNA extraction.

### 4.5. qRT-PCR Analysis

qRT-PCR was performed to analyze the expression profiles of the inducible TRP genes after the chronic heat stress. Total RNA was isolated from each sample with Trizol Reagent (Invitrogen) according to the manufacturer’s instructions. The quantity and quality of total RNA were assessed using NanoDrop (Thermo Fisher Scientific, Wilmington, DE, USA) and 1.2% agarose gel electrophoresis, respectively. cDNA was synthesized from RNA using PrimeScript™ RT reagent Kit with gDNA Eraser (TaKaRa, Dalian, Liaoning, China). Specific primers used in this study were designed in GenScript (https://www.genscript.com/tools/real-time-pcr-taqman-primer-design-tool, accessed on 3 March 2021) (Supplementary Table S4). In addition, elongation factor 1 alpha (EF1α) was used as internal control to normalize expression among various tissues. The PCR reactions were performed on the LightCycler 480 real-time PCR instrument (Roche Diagnostics, Burgess Hill, UK) using SYBR Premix Ex Taq™ (TaKaRa). Cycling parameters were 95 °C for 5 min, then 40 cycles of 95 °C for 5 s, 60 °C for 20 s, and 72 °C for 30 s. All qRT-PCRs were performed in triplicate and a dissociation curve was conducted to confirm amplification specificity. The relative expression analysis was calculated using the comparative *C*_t_ (2^−∆∆*C*t^) method [55]. The raw data of qRT-PCR were provided in Supplementary Table S5.

### 4.6. Statistical Analysis

Significant differences of gene expression among experimental groups were analyzed using a two-way ANOVA followed by Tukey’s multiple comparison test. Values of relative expression were shown as mean ± standard error of the mean (SEM) (*n* = 3). Statistical significance was set as *p* < 0.05.

## 5. Conclusions

We systematically identified and annotated a complete set of 66 TRP genes in *C. gigas*, which were assigned into TRPA, TRPC, TRPM, and TRPV subfamilies. Transcriptome profiling of these TRPs after acute heat stress revealed several heat stress inducible genes, which were further validated in a chronic heat stress experiment. Notably, expression profiles of these heat inducible TRP genes compared between *C. gigas* and *C. angulate*, two species with contrasted tolerance to high temperature stress, allowed identification of TRPC3 and TRPV4 that were involved in thermal regulation toward heat tolerance. This work provided valuable information for future investigations on the molecular mechanism of TRP mediated thermal tolerance, and identification of diagnostic biomarker for thermal stress in the oysters.

## Figures and Tables

**Table 1:** Copy Numbers of TRP Genes in *C. gigas* and Other Representative Species. Information of TRP Genes from Several Other Representative Vertebrate and Invertebrate Species Were Retrieved from NCBI Database (Assembly: cgigas_uk_roslin_v1, with accession of GCA_902806645.1). Abbreviations: Hsa, *Homo sapiens*; Mmu, *Mus musculus*, Gga, *Gallus gallus*; Xtr, *Xenopus tropicalis*; Dre, *Danio rerio*; Cin, *Ciona intestinalis*; Mye, *Mizuhopecten yessoensis*; Cvi, *Crassostrea virginica*; and Cgi, *Crassostrea gigas*.

Gene	Hsa	Mmu	Gga	Xtr	Dre	Cin	Mye	Cvi	Cgi
TRPA1	1	1		1	2	9	5	6	3
TRPC1	1	1	1	1	1				
TRPC2		1		1	1				
TRPC3	1	1	1	1	2	1	4	6	7
TRPC4	1	1	1	1	2			1	
TRPC5	1	1	1	1	1				
TRPC6	1	1	1	1	2				
TRPC7	1	1	1		2				
TRPM1	1	1	1	1	3	1	2	3	5
TRPM2	1	1	1		1	1	5	18	21
TRPM3	1	1	1	1	1		5	5	1
TRPM4	1	1		1	3			1	
TRPM5	1	1	1		1		6	1	
TRPM6	1	1	1	1	1		1		
TRPM7	1	1	1	2	1				1
TRPM8	1	1	1	1			1	5	5
TRPN					1				
TRPV1	1	1	1	1	1		2		4
TRPV2	1	1	1	1					
TRPV3	1	1	1	1				2	
TRPV4	1	1	1	3	1				8
TRPV5	1	1		1		1	4	5	6
TRPV6	1	1	1	2	1		4		

## Data Availability

All the data used in this study have been provided in the main text and Supplementary Materials.

## Supplementary Materials

The following are available online at https://www.mdpi.com/1422-0067/22/6/3222/s1.

## Abbreviations

TRP	transient receptor potential
ROS	reactive oxygen species
H_2_O_2_	hydrogen peroxide
FPKM	fragments Per Kilobase of transcript per Million mapped reads
EF1α	elongation factor 1 alpha
SEM	standard error of the mean
UTRs	untranslated regions
HIF-1α	hypoxia inducible factor 1 α
CAT	catalase
SOD	superoxide dismutase
CAS	caspase

## References

[1] Cheng L., Abraham J., Trenberth K.E., Fasullo J., Boyer T., Locarnini R., Zhang B., Yu F., Wan L., Chen X. (2021). Upper Ocean Temperatures Hit Record High in 2020. Adv. Atmos. Sci..

[2] Khan F.U., Hu M., Kong H., Shang Y., Wang T., Wang X., Xu R., Lu W., Wang Y. (2020). Ocean acidification, hypoxia and warming impair digestive parameters of marine mussels. Chemosphere.

[3] Coen L.D., Bishop M.J. (2015). The ecology, evolution, impacts and management of host–parasite interactions of marine molluscs. J. Invertebr. Pathol..

[4] Guo X., Wang Y., Wang L., Lee J.-H., Kocher T., Kole C. (2008). Oysters. Genome Mapping and Genomics in Fishes and Aquatic Animals.

[5] Cheney D., Elston R., Macdonald B., Kinnan K., Suhrbier A. (2001). The roles of environmental stressors and culture methods on the summer mortality of the Pacific oyster *Crassostrea gigas*. J. Shellfish Res..

[6] Samain J.F., Dégremont L., Soletchnik P., Haure J., Bédier E., Ropert M., Moal J., Huvet A., Bacca H., Wormhoudt A.V. (2007). Genetically based resistance to summer mortality in the Pacific oyster (*Crassostrea gigas*) and its relationship with physiological, immunological characteristics and infection processes. Aquaculture.

[7] Soletchnik P., Ropert M., Mazurié J., Fleury P.G., Coz F.L. (2007). Relationships between oyster mortality patterns and environmental data from monitoring databases along the coasts of France. Aquaculture.

[8] Guo X., He Y., Zhang L., Lelong C., Jouaux A. (2015). Immune and stress responses in oysters with insights on adaptation. Fish Shellfish Immunol..

[9] Lang R.P., Bayne C.J., Camara M.D., Cunningham C., Jenny M.J., Langdon C.J. (2009). Transcriptome Profiling of Selectively Bred Pacific Oyster *Crassostrea gigas* Families that Differ in Tolerance of Heat Shock. Mar. Biotechnol..

[10] Zhang G., Li L., Meng J., Qi H., Qu T., Xu F., Zhang L. (2016). Molecular Basis for Adaptation of Oysters to Stressful Marine Intertidal Environments. Annu. Rev. Anim. Biosci..

[11] Zhang G., Fang X., Guo X., Li L., Luo R., Xu F., Yang P., Zhang L., Wang X., Qi H. (2012). The oyster genome reveals stress adaptation and complexity of shell formation. Nature.

[12] Venkatachalam K., Montell C. (2007). TRP Channels. Annu. Rev. Biochem..

[13] Cosens D.J., Manning A. (1969). Abnormal Electroretinogram from a Drosophila Mutant. Nature.

[14] Fernández-Carvajal A., Fernández-Ballester G., González-Muñiz R., Ferrer-Montiel A., Madrid R., Bacigalupo J. (2015). Pharmacology of TRP Channels. TRP Channels in Sensory Transduction.

[15] Li M., Yu Y., Yang J., Islam M.d.S. (2011). Structural Biology of TRP Channels. Transient Receptor Potential Channels.

[16] Vetter I., Lewis R.J., Islam M.d.S. (2011). Natural Product Ligands of TRP Channels. Transient Receptor Potential Channels.

[17] Cohen M.R., Moiseenkova-Bell V.Y. (2014). Structure of Thermally Activated TRP Channels. Current Topics in Membranes.

[18] Hara Y., Wakamori M., Ishii M., Maeno E., Nishida M., Yoshida T., Yamada H., Shimizu S., Mori E., Kudoh J. (2002). LTRPC2 Ca2+-Permeable Channel Activated by Changes in Redox Status Confers Susceptibility to Cell Death. Mol. Cell.

[19] Nazıroğlu M., Lückhoff A. (2008). A Calcium Influx Pathway Regulated Separately by Oxidative Stress and ADP-Ribose in TRPM2 Channels: Single Channel Events. Neurochem. Res..

[20] Carrasco C., Naziroglu M., Pecze L., Pariente J.A. (2018). Editorial: Involvements of TRP Channels and Oxidative Stress in Pain. Front. Physiol..

[21] Castillo K., Diaz-Franulic I., Canan J., Gonzalez-Nilo F., Latorre R. (2018). Thermally activated TRP channels: Molecular sensors for temperature detection. Phys. Biol..

[22] Huang Y., Roth B., Lü W., Du J. (2019). Ligand recognition and gating mechanism through three ligand-binding sites of human TRPM2 channel. eLife.

[23] Qiu J., Zhang C., Borgquist A., Nestor C.C., Smith A.W., Bosch M.A., Ku S., Wagner E.J., Rønnekleiv O.K., Kelly M.J. (2014). Insulin Excites Anorexigenic Proopiomelanocortin Neurons via Activation of Canonical Transient Receptor Potential Channels. Cell Metab..

[24] Chrétien C., Fenech C., Liénard F., Grall S., Chevalier C., Chaudy S., Brenachot X., Berges R., Louche K., Stark R. (2017). Transient Receptor Potential Canonical 3 (TRPC3) Channels Are Required for Hypothalamic Glucose Detection and Energy Homeostasis. Diabetes.

[25] Farcy É., Voiseux C., Lebel J.-M., Fiévet B. (2009). Transcriptional expression levels of cell stress marker genes in the Pacific oyster *Crassostrea gigas* exposed to acute thermal stress. Cell Stress Chaperones.

[26] Kim B.-M., Kim K., Choi I.-Y., Rhee J.-S. (2017). Transcriptome response of the Pacific oyster, *Crassostrea gigas* susceptible to thermal stress: A comparison with the response of tolerant oyster. Mol. Cell. Toxicol..

[27] Zhu Q., Zhang L., Li L., Que H., Zhang G. (2016). Expression Characterization of Stress Genes Under High and Low Temperature Stresses in the Pacific Oyster, *Crassostrea gigas*. Mar. Biotechnol..

[28] Li A., Li L., Song K., Wang W., Zhang G. (2017). Temperature, energy metabolism, and adaptive divergence in two oyster subspecies. Ecol. Evol..

[29] Wang J., Li Q., Kong L. (2010). Genetic variation and differentiation in wide ranging populations of razor clam (*Sinonovacula constricta*) inferred from AFLP markers. J. Ocean Univ. China.

[30] Patapoutian A., Peier A.M., Story G.M., Viswanath V. (2003). ThermoTRP channels and beyond: Mechanisms of temperature sensation. Nat. Rev. Neurosci..

[31] Boudreaux M.L., Walters L.J., Rittschof D. (2009). Interactions Between Native Barnacles, non-native Barnacles, And The Easternrnoyster *Crassostrea virginica*. Bull. Mar. Sci..

[32] Montell C. (2005). The TRP Superfamily of Cation Channels. Sci. Signal..

[33] Wes P.D., Chevesich J., Jeromin A., Rosenberg C., Stetten G., Montell C. (1995). TRPC1, a human homolog of a Drosophila store-operated channel. Proc. Natl. Acad. Sci. USA.

[34] Ghaffari H., Wang W., Li A., Zhang G., Li L. (2019). Thermotolerance Divergence Revealed by the Physiological and Molecular Responses in Two Oyster Subspecies of *Crassostrea gigas* in China. Front. Physiol..

[35] Kiselyov K., Xu X., Mozhayeva G., Kuo T., Pessah I., Mignery G., Zhu X., Birnbaumer L., Muallem S. (1998). Functional Interaction between InsP 3 Receptors and Store-Operated Htrp3 Channels. Nature.

[36] Guévélou E., Huvet A., Sussarellu R., Milan M., Guo X., Li L., Zhang G., Quillien V., Daniel J.-Y., Quéré C. (2013). Regulation of a Truncated Isoform of AMP-Activated Protein Kinase α (AMPKα) in Response to Hypoxia in the Muscle of Pacific Oyster *Crassostrea gigas*. J. Comp. Physiol. B.

[37] Kim E.-Y., Choi Y.H. (2019). Regulation of Adductor Muscle Growth by the IGF-1/AKT Pathway in the Triploid Pacific Oyster *Crassostrea gigas*. Fish Aquatic. Sci..

[38] Güler A.D., Lee H., Iida T., Shimizu I., Tominaga M., Caterina M. (2002). Heat-Evoked Activation of the Ion Channel, TRPV4. J. Neurosci..

[39] Watanabe H., Davis J.B., Smart D., Jerman J.C., Smith G.D., Hayes P., Vriens J., Cairns W., Wissenbach U., Prenen J. (2002). Activation of TRPV4 Channels (hVRL-2/mTRP12) by Phorbol Derivatives. J. Biol. Chem..

[40] Ely B.R., Lovering A.T., Horowitz M., Minson C.T. (2014). Heat acclimation and cross tolerance to hypoxia: Bridging the gap between cellular and systemic responses. Temp. Multidiscip. Biomed. J..

[41] Lushchak V.I. (2011). Environmentally induced oxidative stress in aquatic animals. Aquat. Toxicol..

[42] Meistertzheim A.L., Tanguy A., Moraga D., Thébault M.T. (2007). Identification of differentially expressed genes of the Pacific oyster *Crassostrea gigas* exposed to prolonged thermal stress. FEBS J..

[43] Özkaya D., Nazıroğlu M. (2020). Curcumin diminishes cisplatin-induced apoptosis and mitochondrial oxidative stress through inhibition of TRPM2 channel signaling pathway in mouse optic nerve. J. Recept. Signal Transduct..

[44] Heise K., Puntarulo S., Nikinmaa M., Lucassen M., Pörtner H.-O., Abele D. (2006). Oxidative stress and HIF-1 DNA binding during stressful cold exposure and recovery in the North Sea eelpout (*Zoarces viviparus*). Comp. Biochem. Physiol. Part A Mol. Integr. Physiol..

[45] Heise K., Puntarulo S., Nikinmaa M., Abele D., Pörtner H.-O. (2006). Oxidative stress during stressful heat exposure and recovery in the North Sea eelpout *Zoarces viviparus* L.. J. Exp. Biol..

[46] Klumpen E., Hoffschröer N., Zeis B., Gigengack U., Dohmen E., Paul R.J. (2017). Reactive oxygen species (ROS) and the heat stress response of Daphnia pulex: ROS-mediated activation of hypoxia-inducible factor 1 (HIF-1) and heat shock factor 1 (HSF-1) and the clustered expression of stress genes. Biol. Cell.

[47] Rissanen E. (2006). Temperature regulates hypoxia-inducible factor-1 (HIF-1) in a poikilothermic vertebrate, crucian carp (*Carassius carassius*). J. Exp. Biol..

[48] Altschul S. (1997). Gapped BLAST and PSI-BLAST: A new generation of protein database search programs. Nucleic Acids Res..

[49] Chen C., Chen H., Zhang Y., Thomas H.R., Frank M.H., He Y., Xia R. (2020). TBtools: An Integrative Toolkit Developed for Interactive Analyses of Big Biological Data. Mol. Plant.

[50] Katoh K., Standley D.M. (2013). MAFFT Multiple Sequence Alignment Software Version 7: Improvements in Performance and Usability. Mol. Biol. Evol..

[51] Kalyaanamoorthy S., Minh B.Q., Wong T.K.F., von Haeseler A., Jermiin L.S. (2017). ModelFinder: Fast model selection for accurate phylogenetic estimates. Nat. Methods.

[52] Nguyen L.-T., Schmidt H.A., von Haeseler A., Minh B.Q. (2015). IQ-TREE: A Fast and Effective Stochastic Algorithm for Estimating Maximum-Likelihood Phylogenies. Mol. Biol. Evol..

[53] Louis A., Muffato M., Roest Crollius H. (2012). Genomicus: Five genome browsers for comparative genomics in eukaryota. Nucleic Acids Res..

[54] Pertea M., Kim D., Pertea G.M., Leek J.T., Salzberg S.L. (2016). Transcript-level expression analysis of RNA-seq experiments with HISAT, StringTie and Ballgown. Nat. Protoc..

[55] Livak K.J., Schmittgen T.D. (2001). Analysis of relative gene expression data using real-time quantitative PCR and the 2(−Delta Delta C(T)) Method. Methods.

